# Percolation-Driven NO_2_ Sensing in Structurally Tuned Sn/SnO Nanoparticles at Room Temperature with Parts-per-Billion Sensitivity

**DOI:** 10.3390/s26092651

**Published:** 2026-04-24

**Authors:** Wilfredo Otaño, Adrian Camacho, Wilanyi Alvarez, Wanda Rivera, Francisco Bezares, Danilo Barrionuevo, Victor M. Pantojas

**Affiliations:** 1 Department of Mathematics-Physics, University of Puerto Rico at Cayey, Cayey 00736, Puerto Rico; wilfredo.otano@upr.edu (W.O.); wilanyi.alvarez@upr.edu (W.A.); wanda.rivera17@upr.edu (W.R.); danilo.barrionuevo@upr.edu (D.B.); 2 Venus Sensors LLC, San Juan 00921, Puerto Rico; 3 Department of Chemistry and Physics, University of Puerto Rico at Ponce, Ponce 00732, Puerto Rico; adrian.camacho@upr.edu; 4 Department of Physics, University of Puerto Rico at Mayaguez, Mayaguez 00680, Puerto Rico; francisco.bezares1@upr.edu

**Keywords:** chemiresistive gas sensors, nanotechnology, tin oxides, Romarchite, nanograins, nitrogen dioxide, core–shell nanograins

## Abstract

**Highlights:**

**What are the main findings?**

- A novel percolation-driven sensing mechanism in Sn/SnO nanostructures at room temperature is identified.
- Tin- and tin oxide-based sensors are ultra-sensitive to NO_2_, with the lowest tested concentration of 100 parts-per-billion at room temperature.

**What are the implications of the main findings?**

- The sensor demonstrates parts-per-billion sensitivity while operating at room temperature.
- Sensitivity can be further improved by controlling the particle size in percolation-driven gas sensing.

**Abstract:**

Monitoring air quality is crucial for understanding and improving public health. There is interest in developing ultra-sensitive, low-power, cost-effective sensors. This work demonstrates that structural modulation of Sn nanoparticles through controlled deposition and oxidation enables a transition from metallic to semiconducting percolative networks, significantly enhancing NO_2_ sensing performance at room temperature. The proposed percolation-driven sensing mechanism provides a new framework for understanding charge transport and gas interaction in nanostructured metal oxide systems. The nanoparticles are deposited near the percolation threshold for electrical conduction and, upon exposure to air, consist of a tin core and an amorphous Sn_3_O_4_ surface. Post-deposition heating in air at 320 °C for two hours forms SnO and Sn_3_O_4_ on top of the gold electrodes and polycrystalline SnO in the tetragonal litharge phase, known as Romarchite, on the glass between the electrodes. Both as-deposited and heat-treated sensors were capable of detecting NO_2_ at room temperature, with a limit of detection in the parts-per-billion range. A percolation model is used to explain their operating currents, in which NO_2_ reacts at nanoparticle gaps and intra-grain boundaries to form charge-depletion regions that primarily determine their resistance. Heat treatment has also been found to cause disproportionation of SnO, resulting in tin-rich precipitates and increasing the operating current to the milliampere range. These precipitates, although oxidized on their surfaces when exposed to air, may serve as bridges that reduce the total resistance of the percolating paths.

## 1. Introduction

Assessing air quality is essential for communities worldwide. Monitoring air quality is vital for public health. The Lancet Commission reported that pollution causes about 9 million deaths per year globally [1]. Harmful outdoor gases like NO_2_ and CO are linked to various heart and lung diseases [2,3]. The World Bank estimated that air pollution cost $8.1 trillion in 2019 [4]. Air monitoring separates harmful particulates from gases. Sophisticated instruments capable of differentiating gases are costly and require expensive maintenance, such as gas chromatography/mass spectrometry and photoionization detectors [5,6,7]. Developing low-cost, adaptable sensors is desirable. Features might include ultra-sensitivity (parts per billion, ppb), IoT connectivity, portability, ease of use, and geospatial support. Room-temperature operation and low power consumption are also beneficial, especially for field stations. These sensors could benefit remote communities, resource-limited regions, and diverse geospatial areas.

Several groups have reported room-temperature gas sensing on both rigid and flexible substrates, with a limit of detection (LOD) in the low-ppb range [8,9,10,11,12,13,14,15,16,17]. These promising technologies still need to demonstrate successful commercial implementation, as they often require numerous processing steps, expensive materials, or are challenging to integrate with low-cost IoT-enabled stations. Metal oxide (MO) gas sensors are studied because of their sensitivity, stability, and low cost [15,18,19,20,21,22]. Furthermore, studies by leading research groups have demonstrated the critical role of nanostructured metal oxides in achieving high-performance NO_2_ detection [23,24]. These works highlight the role of surface chemistry, defect engineering, and nanostructuring in enhancing sensitivity and selectivity.

Knowledge of the formation of MO materials with diverse morphologies, especially at the nanometer scale, has driven the development of chemiresistive sensors, such as ultrathin films, nanowires, and nanoparticles, which offer large surface areas for reactions while requiring less material [18,25]. These sensors typically operate at higher temperatures, which makes them unsuitable for IoT and portable device applications [7,26]. Therefore, MO sensors, which operate at room temperature and consume low power, are promising for low-cost air quality monitoring stations.

This work describes chemiresistive metal-oxide sensor elements made of tin nanoparticles deposited on commercial interdigitated electrodes (IDEs). The deposition time was selected so that the material is near the percolation threshold for electrical conduction. The nanoparticles have a tin core and an amorphous Sn_3_O_4_ surface that forms upon exposure to air. After post-deposition oxidation, they transform into polycrystalline SnO in the tetragonal litharge phase, known as Romarchite. Both compositions, with and without post-deposition oxidation, are evaluated as sensing elements for NO_2_ detection at room temperature. They are easy to construct, have low power consumption, use a small amount of low-cost material, and integrate easily into platforms for the most stringent applications.

## 2. Materials and Methods

### 2.1. Synthesis of Tin Nanostructures

The initial step in the process involves forming a tin metal nanostructure on the interdigitated areas of the IDEs. A commercial tin metal target (99.99%) with a 5.08 cm diameter is magnetron sputtered in an argon plasma at 1.33 Pa using 50 W of DC power. The target-substrate distance was fixed at 69 mm, and the background pressures were below 1.33 × 10^−4^ Pa. The metal is deposited on commercial IDEs (Micrux Technologies, Gijon, Spain) with five-micrometer-wide interdigitated gold electrodes separated by the same distance. A mask prevents tin deposition on the gold pads used as electrical contacts. The samples were heated at various temperatures during deposition, reaching up to 250 °C, using radiant lamps. A thermocouple, mechanically attached to the holder, was used to measure the deposition temperature. Deposition time is a critical parameter in sensor fabrication, as it directly dictates the morphology, density, and electrical connectivity of the sensing material. The deposition time and substrate temperature were controlled parameters, while plasma pressure, sputtering power, and target-to-substrate distance were kept constant. The second step of the process involves heating the deposited IDEs in a conventional open-air oven at 320 °C for 2 h to form the tin monoxide phase.

### 2.2. Material Characterization

The sensing materials were studied using X-ray diffraction (XRD), scanning electron microscopy (SEM), transmission electron microscopy (TEM), and Raman spectroscopy. The XRD characterization was performed on a Rigaku SmartLab (Rigaku Corporation, Tokyo, Japan) system equipped with a HyPix-300 detector (Rigaku Corporation) and sample-heating capabilities using a parallel-beam configuration with Cu K-alpha radiation at 40 kV and 40 mA, a step size of 0.02°, and a scanning speed of 2°/min.

The samples prepared for TEM measurements were deposited at 150 °C for various sputtering times onto a 10 nm-thick hexagonal-shaped frame of silicon nitride TEM windows, purchased from SiMPore (SiMPore Corporation, West Henrietta, NY, USA). The TEM analysis was performed using a JEOL F200 (JEOL Ltd., Tokyo, Japan) at the Singh Center for Nanotechnology at the University of Pennsylvania. Raman spectroscopy was performed using a JY-Horiba 64000 spectrometer (HORIBA S.A.S., Grabels, France) equipped with a Triple monochromator in subtractive mode, a CCD detector, and an Olympus microscope with an 80× objective in backscattering mode. The laser is a Coherent Argon Innova 70C (Coherent Corporation, Santa Clara, CA, USA), operating at 514.53 nm with 5 mW of power on the sample.

### 2.3. Sensing Measurements

**Figure 1:**
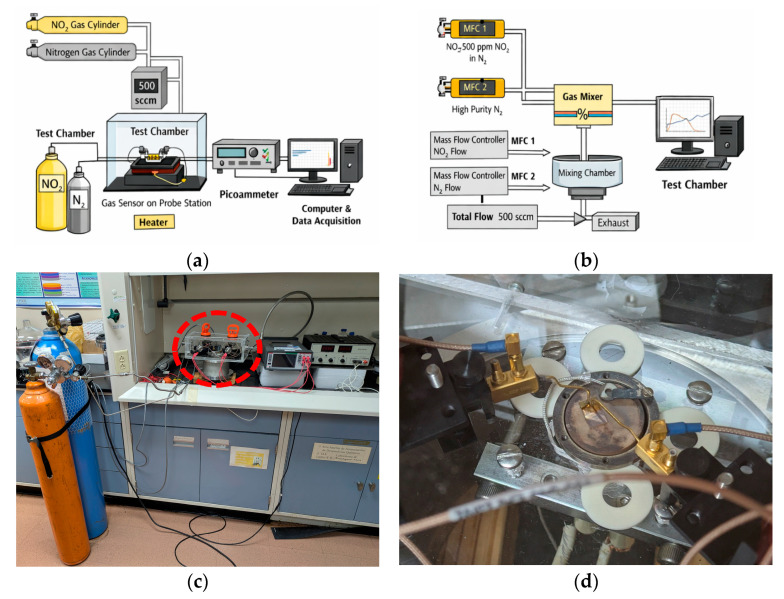
The gas sensing system consist of a gas cylinders, mass flow controller, test chamber with the sensor element mounted on a probe station, and electrical measurements using a picoammeter connected to a computer for real-time data acquisition. (**a**) Experimental set-up. (**b**) Gas flow system. (**c**) Photo of the experimental station, the red circle indicates the test chamber. (**d**) Photo of the test chamber, gold probes make contacts to IDE from the top.

The material was tested as a gas sensor in a commercial probe station (MMR Technologies LTMP, San Jose, CA, USA). A Keithley 6487 picoammeter/voltage source (Keithley Instruments Inc., Cleveland, OH, USA) applied a constant bias voltage of 1–5 V across the IDEs while measuring the current. An MKS mass flow controller (MKS Instruments, Inc., Wilmington, MA, USA) was used to set the total gas flow to 500 sccm, while using N_2_ as the carrier gas. The gas exposure was controlled manually through mass flow controllers, which may introduce minor uncertainties in switching time and gas stabilization. However, the total flow rate (500 sccm) and chamber volume ensure rapid gas exchange, minimizing transient delays. The estimated uncertainty in gas exposure timing is on the order of a few seconds, which does not significantly affect the observed sensing trends. A schematic diagram of the experimental setup is shown in Figure 1. Although the system was not fully automated, LabVIEW software (LabView 2010) was used to record time-resolved current (I vs. t) responses. At the same time, the mass flow controllers were manually operated to deliver and stop gas flow.

## 3. Results

### 3.1. Tin Nanoparticles

#### 3.1.1. Characterization of Tin Nanoparticles

**Figure 2:**
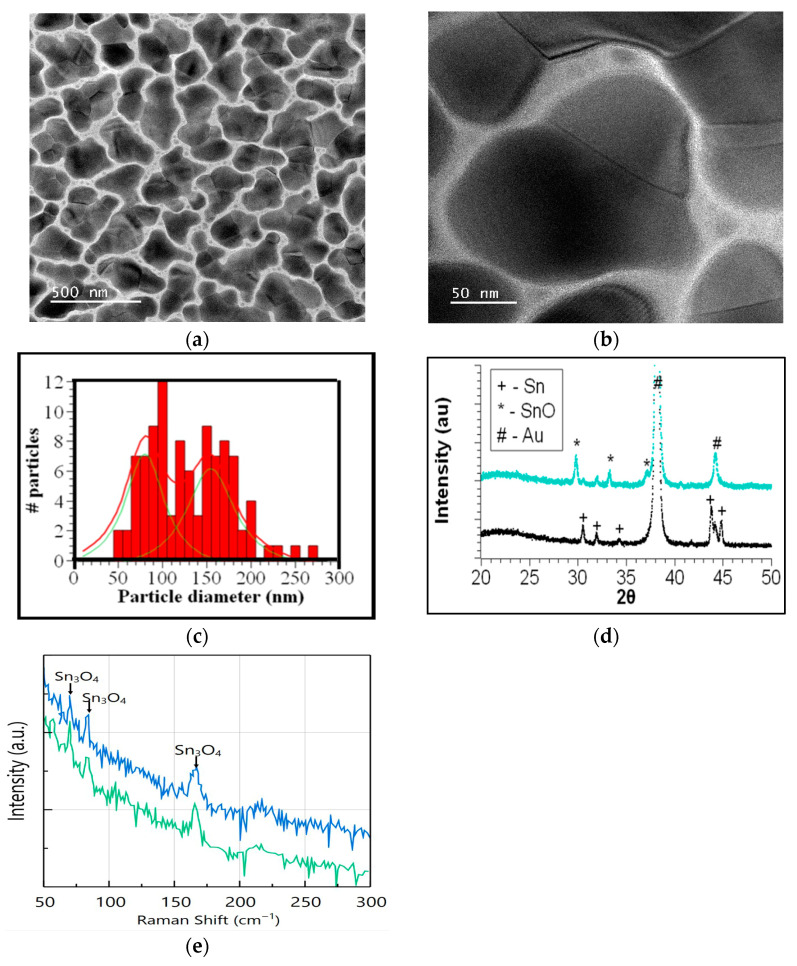
(**a**) TEM of a sample deposited on a silicon nitride membrane at 150 °C for 15 s. (**b**) TEM at higher magnification, showing the juxtaposition of grains to form a large particle. (**c**) Frequency distribution of the particle diameter. The distribution can be fitted with a bimodal distribution (red line) deconvoluted into two distinct component distributions shown in green. (**d**) XRD of a sample deposited on an IDE at 150 °C for 130s (black) and after heat treatment at 320 °C for two hours in air (cyan). The asterisk identifies peaks corresponding to the Romarchite phase of SnO that forms after heat treatment. (**e**) Raman spectra of Sn deposited on IDE. In blue, measured at the glass region between interdigitated electrodes and in green at the gold pad region.

The physical vapor deposition of tin on weakly interacting substrates occurs in three stages: pre-nucleation, nucleation, and agglomeration. For chemiresistor sensors, the agglomeration stage is crucial for forming percolation paths that enable current to flow. TEM measurements provide insights into the morphology of Sn metal agglomerates. Figure 2a,b shows TEM images at two magnifications of a sample deposited for 15 s. Island agglomerates range in size from a few nanometers to over 200 nanometers and exhibit distinctive grain boundaries. The minimum, maximum, and average diameters were obtained from measurements on 100 particles larger than 50 nm due to imaging limitations and contrast considerations. Smaller particles (<50 nm) were observed but not included in the statistical analysis. A frequency distribution of particle sizes is shown in Figure 2c, fitted with a convolution of two Lorentzian functions with peaks at 80 and 155 nm. A similar bimodal distribution was reported by Martinez Medina et al. in their study of coalescence in clusters of nanostructured Sn films, which they attributed to diffusion and juxtaposition [27]. Using high-resolution TEM, they identified interfaces between clusters, like those shown in Figure 2a, consistent with previous findings by Hishita et al. [28] for Sn evaporated on glass. Martinez Medina et al. [27] observed that these interfaces disappeared and proposed a mechanism of cluster recrystallization to explain this phenomenon. This mechanism accounts for the nanograins seen in Figure 2a, where the homologous temperature Ts/Tm is 0.84 for a substrate heated to Ts = 423 K and a Sn melting temperature Tm = 505 K. High surface diffusion on a weakly interacting substrate favors the formation of individual grains that eventually coalesce due to melting at the interface. Therefore, larger grains within the size distribution can be understood as a juxtaposition of smaller ones from the smaller distribution. Another size distribution centered at about 25 nm corresponds to the smallest particles surrounding the larger ones.

The XRD of a sample deposited at 150 °C for 130 s is shown in Figure 2d. Five peaks corresponding to beta Sn and two from the gold-interdigitated electrodes are identified. It is well known that exposing 3D tin metal grains to air forms an amorphous oxide layer at the surface, resulting in a core–shell structure [29]. Raman spectroscopy was used to identify the amorphous phases present. As shown in Figure 2e, the Raman spectra of the tin clusters deposited on the glass and gold pad regions exhibit three peaks characteristic of the Sn_3_O_4_ phase. These results indicate that exposing nanoparticles to air after removal from the vacuum chamber results in surface oxidation.

#### 3.1.2. Evaluation of Tin-Based Sensor Prototype

**Figure 3:**
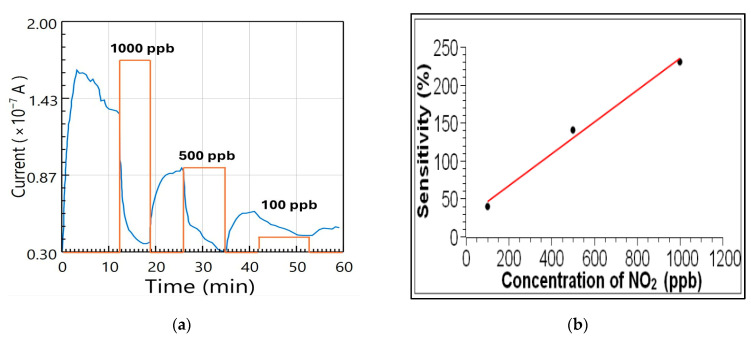
(**a**) Measurement of the SnO sensor current as a function of time. The orange line serves as a visual aid to indicate when NO_2_ is introduced and the corresponding amount. (**b**) Sensor sensitivity as a function of the NO_2_ concentration in parts-per-billion. The red line is a visual aid.

The sensor response was investigated by measuring the current in the IDEs as NO_2_ gas was introduced into the N_2_ flow. The concentrations were chosen to be 1000, 500, and 100 ppb, as shown in Figure 3a. As expected for an oxidizing gas, the current decreases when NO_2_ is introduced and increases when it is cut off. The decrease in current during the initial N_2_ flow was estimated from points on the curve just before the gas introduction, and the response current was adjusted accordingly. Reducing the concentration of NO_2_ decreases the change in current, Δ*I* = |*I*_m_ − *I*_b_|, where *I*_b_ is the base current when the N_2_ is flowing in the testing chamber, and *I*_m_ is the current when the NO_2_ is added. The sensitivity of the sensor is calculated with the following equation:

(1) $$Sensitivity\;\left(\%\right)=\frac{\left|I_{m}-I_{b}\right|}{I_{m}}\;\times \;100.$$

Figure 3b shows the sensitivity plot for the three concentrations. The sensitivity increases from 30% at 100 ppb to 230% at 1000 ppb. The dependence of the sensitivity on concentration can be approximated by a linear fit in the range 100–1000 ppb of NO_2_, as illustrated in the figure for reference. This line serves only as a visual guide, and no quantitative calibration can be established from the present data due to the limited dataset. Additional measurements are currently being performed to improve statistical reliability. Preliminary repeatability tests indicate consistent trends in sensor response; however, future work will include multiple datasets with error bars to quantify reproducibility and uncertainty.

The extent of the decrease in base current cannot be explained solely by the adsorption of NO_2_ on the Sn shell surface. Since NO_2_ is an oxidizing gas, its adsorption on the surface should lead to a reduction in the free electron density of the tin metal, according to:

(2) $$NO_{2(phys)}+{\left(e^{-}\right)}_{CB}\to NO_{2(ads)}^{-}$$

This reaction would reduce the electron current in the sensor, albeit only slightly. For example, a rough estimate of the number of electron carriers in a 50 nm spherical grain of tin with an electron density of 2.55 × 10^28^/cm^3^ is approximately 10^7^ electrons. For the same-size grain, if atoms in the surface correspond equally to the two distinct faces of the body-centered tetragonal unit cell, with areas A_1_ = a^2^ and A_2_ = a·c, where a = 583 pm and c = 318 pm, then the average surface area of the unit cell is 2.62 × 10^−19^ m^2^. Only ¼ of an atom occupies this area; therefore, the estimated number of atoms on the surface of the 50 nm grain, with a surface area of $7.85\times 10^{-15}$ m^2^, is

(3) $$\#atoms=\frac{1}{4}at\times \frac{7.85\times 10^{-15}}{2.62\times 10^{-19}}=7.49\times 10^{3}\mathrm{atoms}.$$

Even if the NO_2_ molecule takes one electron from each surface Sn atom, the resulting change in sensor current should be less than 1%.

#### 3.1.3. Percolation in the Tin Sensor

**Figure 4:**
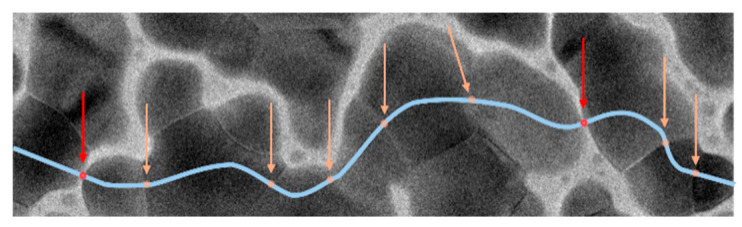
Possible percolation path for current (in light blue) with high resistivity regions from gaps between grains (in red) and grain boundaries (light orange).

A percolation model for conductivity in the sensor is proposed to account for the larger-than-expected change in current when exposed to NO_2_. The model is supported by examining the morphology of the agglomerated nanograins. As shown in Figure 4, current can percolate along paths between the gold electrodes, passing through particle gaps and intragrain boundaries. The total resistance of the percolating path could be described as the sum of the resistances of three representative regions: R1 is the resistance within the tin nanograin, R2 is the resistance between two nanograins, and R3 is the resistance across a grain boundary. The total resistance of the percolative path is

(4) $$R_{T}=\sum R1+\sum R2+\sum R3≅\sum R2.$$

The current in the percolative path is dictated by the highest resistances (R2 and R3) since R2 ≥ R3 >> R1. These high-resistivity regions determine the sensor’s conductance to be in the 10^−7^ A range. After NO_2_ is introduced, the total resistance increases because the oxidizing gas is absorbed into the nanograins. Evidence for this is that the initial sensor response to NO_2_ occurs within seconds and is partially reversible, indicating that the reaction initiates at the grain surface and then diffuses to the grain boundaries and the tin core.

Thus, the sensing mechanism is attributed to NO_2_ adsorption at nanoparticle surfaces, grain boundaries, and interparticle gaps. NO_2_ acts as an electron acceptor, creating depletion regions that increase resistance. The percolation model suggests that conduction is dominated by high-resistance regions (grain boundaries and gaps), where gas adsorption significantly alters the transport pathways. The rapid response and partial reversibility indicate surface-driven adsorption followed by diffusion into grain boundaries. This mechanism is consistent with charge depletion models reported for metal oxide gas sensors. While the percolation-based model provides a consistent explanation of the observed sensing behavior, additional experimental validation, such as temperature-dependent conductivity, I–V characterization, and impedance spectroscopy, will be performed in future work to further substantiate this mechanism.

### 3.2. Tin Monoxide Nanoparticles

#### 3.2.1. Characterization of Tin Monoxide Nanoparticles

**Figure 5:**
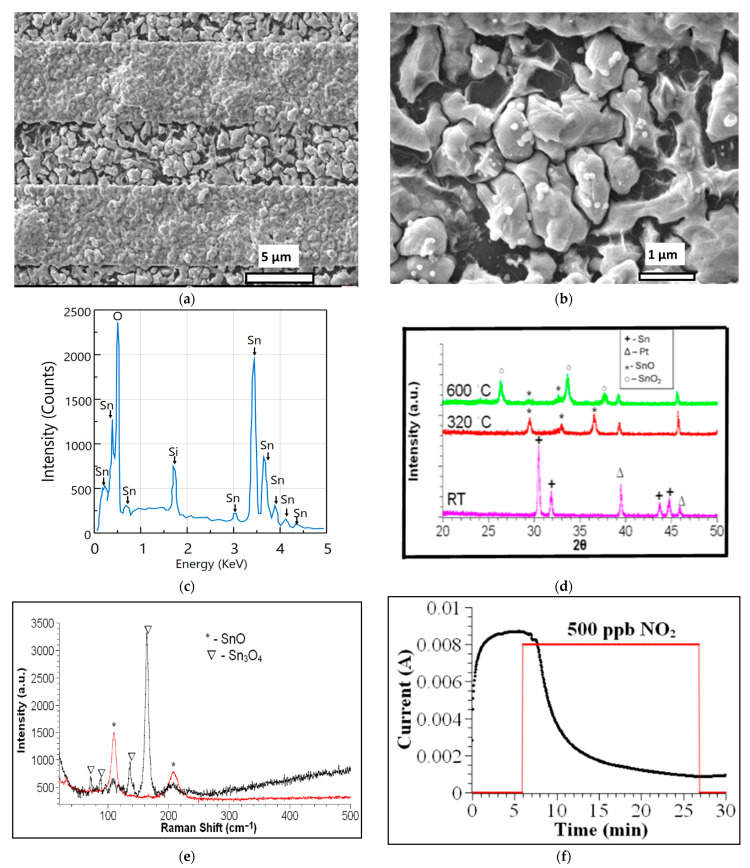
(**a**) SEM micrograph of a sample deposited on an IDE at 150 °C for 30 s, followed by the oxidation process in the conventional oven at 320 °C for two h. (**b**) A magnification of the region between gold pads showing spherical metallic precipitates. (**c**) EDS spectrum of spheroid-like precipitate. (**d**) XRDs show the crystal-line phase transformation of Sn-SnO-SnO_2_ after heat treatment to 320 °C and 600 °C. (**e**) Raman spectra after heat treatment. In red, measured at the glass region between interdigitated electrodes. In black, measured at the gold pad region. (**f**) Graph of current as a function of time as measured by the SnO sensor for 500 ppb of NO_2_. The red line serves as a visual aid, showing the approximate time the NO_2_ gas is introduced and the corresponding amount.

After heating the tin nanoparticles to produce tin oxide, we observe different morphologies on the IDE. Figure 5a,b shows SEM micrographs of a sample deposited at 150 °C for 30 s and subsequently oxidized. The agglomeration on top of the gold interdigitated strips is more pronounced than on the glass, suggesting a stronger bond between the tin and gold.

A magnification of the region deposited on the glass reveals the precipitation of small, spheroid-like material on the surface of the grains, as shown in Figure 5b. The atomic composition of these grains was evaluated using EDS and shown in Figure 5c. Table 1 shows the atomic percentages of Sn, Si, and O. In SiO_2_, there are two oxygen atoms per silicon atom. The oxygen atomic percentage bonded to tin atoms is 50.44 − 2(5.12) = 40.2%, and the fraction of metallic tin in the precipitate is (44.44 − 40.2)/44.44 = 9.54%. These results indicate that the Sn content in these grains exceeds the oxygen content by 9.54%.

These precipitates are observed in all the samples heated at 320 °C for 2 h. Their presence is also detected by XRD, as shown in Figure 2d. Their formation has been associated with the disproportionation of SnO to Sn and Sn_3_O_4_ at temperatures above 250 °C, or with Sn_3_O_4_ disproportionation to SnO_2_ and Sn at temperatures above 200 °C [30,31,32]. The absence of XRD or Raman peaks that correspond to the SnO_2_ phase indicates that they are formed from the disproportionation of SnO.

XRD reveals the formation of the Romarchite phase of SnO (cards 7222460 and 9012005) while peaks of beta-Sn decrease in intensity or vanish after the heat treatment (Figure 2d). The lattice parameters a = 3.80844 Å, b = 3.80844 Å, and c = 4.84636 Å are calculated for the tetragonal structure. Although a full Rietveld refinement was not performed, peak positions and phase identification were consistent with standard crystallographic databases. Romarchite is a layered structure where the s-orbital lone pair stabilizes the antibonding state [32,33].

The evolution of the crystalline phases was studied by performing XRD of samples deposited on silicon substrates and heated to 320 °C and 600 °C. Figure 5d shows the diffractograms, which exhibit peaks for Sn, Pt (from the holder), SnO, and SnO_2_. The temperature sequence illustrates the transformation of tin nanoparticles into SnO as the temperature is increased to 320 °C, followed by the emergence of the SnO_2_ phase at 600 °C. Post-deposition oxidation at 320 °C results in the formation of the distinctive SnO Romarchite phase. Alongside the metallic Sn precipitates and the formation of crystalline SnO, Raman measurements on the gold pads show peaks associated with Sn_3_O_4_ (Figure 5e). The Sn_3_O_4_ phase is amorphous, as indicated by the absence of its characteristic XRD peaks. Thus, the tin-monoxide sensor consists of amorphous tin oxide (Sn_3_O_4_) on top of the gold electrodes and crystalline tin oxide (SnO) mixed with metallic partially oxidized tin precipitates between the electrodes.

#### 3.2.2. Evaluation of Tin Oxide-Based Sensing Element

The SnO sensing element was evaluated using a concentration of 500 ppb of NO_2_ in the flowing gas (Figure 5f). The base current is high, in the mA range, which is five orders of magnitude greater than that of the tin sensor. This appears counterintuitive, as the oxidation of the tin core, converting Sn into SnO, should increase the resistance of the percolative path and thereby decrease the sensor’s current. An explanation is that the spheroid-like precipitates, containing excess tin atoms, can act as bridges between grains, providing a percolative pathway with lower resistance for the percolation current. Substantial changes in specific properties, such as the current, when comparing the tin and tin monoxide structures, are typical of percolative systems. When NO_2_ is introduced into the testing station, the current decreases because the gas is absorbed more readily in the region between grains, where it reacts with the precipitates, thereby increasing the total resistance.

The sensor’s current decreases by 952% relative to the baseline, which is higher than the approximately 140% decrease observed in the sample without post-deposition oxidation. After shutting off the NO_2_ line, the current did not recover, and the sensor did not respond to subsequent NO_2_ cycles, indicating that the molecular attachment was irreversible at room temperature. This irreversible behavior after NO_2_ exposure suggests possible chemical degradation or strong chemisorption. This effect is attributed to structural changes at grain boundaries and requires further investigation.

The interaction between the NO_2_ molecule and SnO is complex and difficult to access. SnO is a p-type semiconductor but can become n-type under specific temperature and oxygen conditions [32]. As observed in our samples, the chemisorption of an oxidizing gas, such as NO_2_, removes electrons, thereby reducing the conductivity of an n-type semiconductor. Several reports find that SnO converts to an n-type semiconductor when part of the material transforms into SnO_2_. The absence of the SnO_2_ phase in the samples suggests that a different mechanism is responsible for the observed reduction in current relative to the Sn_3_O_4_ sample. This result requires further investigation.

## 4. Discussion

All measurements were conducted using N_2_ as a carrier gas to ensure controlled conditions and isolate the intrinsic sensing behavior of the materials. While real-world environments contain oxygen, humidity, and other pollutants, the use of nitrogen allows reproducible baseline measurements. Oxygen is expected to influence adsorption/desorption kinetics and baseline resistance. Humidity is also known to significantly influence gas sensing performance by competing for adsorption sites, often inhibiting the adsorption of the target gas and reducing response. Water at the surface often acts as an electron donor, modifying surface charge states and changing the baseline resistance [34]. Although humidity was not explicitly controlled in this study, it is expected to alter baseline resistance and response magnitude.

Similarly, the selectivity of the sensor toward other oxidizing and reducing gases (e.g., CO, NH_3_, H_2_) has not yet been systematically evaluated. However, based on literature reports for metal oxide sensors, cross-sensitivity is expected. Future work will include testing under ambient air conditions to evaluate performance in realistic environments, as well as the possibility of enhancing selectivity through surface functionalization and doping.

Table 2 compares the sensing performance of the present Sn/SnO-based sensors with recently reported NO_2_ sensors. The results demonstrate that the proposed system achieves competitive ppb-level detection at room temperature without the need for external heating, highlighting its potential for low-power and portable sensing applications. In particular, the percolation-based conduction mechanism provides a distinct advantage in enhancing sensitivity compared to conventional metal oxide sensors.

A few additional observations can be made about the sensors.

- The response time, defined as the time required to reach 90% of the maximum signal change, is in the order of a few seconds for both Sn and SnO sensors. Recovery behavior differs significantly between the two systems: the Sn sensor exhibits partial reversibility, while the SnO sensor shows limited recovery, indicating strong adsorption or irreversible processes.
- The observed baseline drift before gas exposure is attributed to slow surface equilibration processes and possible adsorption/desorption of residual species in the chamber. Additionally, the absence of full saturation suggests that the system may not have reached steady-state conditions during the measurement window.
- The incomplete recovery after NO_2_ exposure in the SnO sensor indicates strong chemisorption or diffusion into grain boundaries, consistent with the proposed percolation model. These effects highlight the complexity of charge transport in nanostructured systems and suggest that further optimization is required for stable and fully reversible sensing.

## 5. Conclusions

The deposition of Sn by dc-magnetron sputtering on weakly interacting surfaces heated to temperatures with Ts/Tm = 0.84 produces 3D grains that eventually merge into larger grains. By choosing the appropriate deposition time, the material can be tuned to be near the percolation threshold for electrical conduction, enhancing the sensitivity to NO_2_ gas. XRD and Raman spectroscopy analyses showed that the grains consist of beta-Sn crystallites surrounded by an oxide layer of Sn_3_O_4_ that forms upon exposure to air.

These tin nanostructures were evaluated as sensing elements to detect NO_2_ gas. The current through the sensor is about 10^−7^ A, decreases within seconds upon exposure to NO_2_, and is only partially reversible. These observations, along with analysis of TEM micrographs, lead the authors to conclude that electrical conduction is controlled by percolation, where regions comprising grain gaps and grain boundaries with surface oxide layers are the dominant high-resistance nanostructures along the percolation path. NO_2_ can more easily diffuse into these regions, reacting with the oxidative layer and thereby increasing the overall resistance. The lowest tested concentration for the tin nanostructures was 100 ppb.

A second NO_2_ sensing element is prepared by oxidizing the tin nanoparticles after deposition. This process forms the tin monoxide phase, known as Romarchite, and produces spheroidal precipitates that contain an excess of metallic tin. The absence of the SnO_2_ phase indicates that the precipitates form from the disproportionation reaction of SnO. The sensor’s baseline current rises by several orders of magnitude compared to the tin sensor, which is counterintuitive because metallic tin should normally be oxidized. A proposed mechanism suggests that spheroid-like precipitates act as bridges, lowering resistance across grain gaps and grain boundaries, which greatly increases the operating current.

The demonstrated room-temperature operation and low power consumption make these sensors suitable for integration into portable and IoT-based air quality monitoring systems. The simple device architecture enables direct integration with printed circuit boards (PCBs) and low-power readout electronics. Additionally, compatibility with microfabrication processes allows for potential incorporation into compact sensing modules for real-time environmental monitoring, as shown in recent system-level implementations proposed by Gao et al. [35].

In conclusion, two sensing elements with complex nanostructures were fabricated by carefully adjusting the plasma sputtering conditions for tin deposition directly on top of IDEs. Both are highly sensitive to NO_2_, detecting concentration in the parts-per-billion range at room temperature. The tin monoxide sensing element has the advantage of operating at higher currents but lacks cyclability. Conversely, the tin sensor is partially reversible, though it operates at currents five orders of magnitude lower. Both are easy to assemble, consume low power, and require only a small amount of inexpensive material.

## Figures and Tables

**Table 1:** Atomic percentages of Sn, Si, and O.

Element	Precipitate (%)
Sn	44.44 ± 0.30
Si	5.12 ± 0.12
O	50.44 ± 0.43

**Table 2:** Comparison of NO_2_ gas sensing performance with recent reports.

Material/Structure (Reference)	Operating Temperature	Detection Limit	Sensitivity /Response	Response Time	Recovery	Key Feature
Sn nanoparticles (this work)	room temperature	100 ppb	30–230% (100–1000 ppb)	seconds	Partial	percolation-driven conduction
SnO nanoparticles (this work)	room temperature	500 ppb	~952%	seconds	Irreversible	High current operation
rGO/CuO nanoflakes [11]	room temperature	~100 ppb	High	Fast	Reversible	Hybrid nanostructure
SnO_2_/ZnO nanofibers [17]	200–300 °C	~500 ppb	High	Fast	Good	Heterostructure enhancement
SnS_2_ nanopetales [13]	room temperature	~10 ppb	Moderate	Moderate	Good	2D morphology
WO_3_/MWCNT composites [10]	room temperature	~20 ppb	High	Fast	Good	CNT-enhanced conductivity
Flexible metal oxide films [16]	room temperature	~50 ppb	Moderate	Fast	Good	Wearable application

## Data Availability

The original contributions presented in this study are included in the article. Further inquiries can be directed at the corresponding author.

## Abbreviations

The following abbreviations are used in this manuscript:

IDEs	Interdigitated electrodes
ppb	parts per billion
XRD	X-ray diffraction
SEM	Scanning electron microscopy
TEM	Transmission electron microscopy.
